# Use of Centrifugal Filter Devices to Concentrate Dengue Virus in Mosquito *per os* Infection Experiments

**DOI:** 10.1371/journal.pone.0138161

**Published:** 2015-09-15

**Authors:** Vaea Richard, Jérôme Viallon, Van-Mai Cao-Lormeau

**Affiliations:** Institut Louis Malardé, Papeete, French Polynesia; Centro de Pesquisas René Rachou, BRAZIL

## Abstract

Dengue virus (DENV) is an arbovirus transmitted to humans by the bite of infected *Aedes* mosquitoes. Experimental *per os* infection of mosquitoes with DENV is usually a preliminary step in virus/vector studies but it requires being able to prepare artificial blood-meals with high virus titers. We report here the convenient use of centrifugal filter devices to quickly concentrate DENV particles in cell-culture supernatants. The median viral titer in concentrated-supernatants was 8.50 log_10_ TCID_50_/mL. By using these DENV concentrated-supernatants to prepare infectious blood-meals in *Aedes aegypti per os* infection experiments, we obtained a mean mosquito-infection rate of 94%. We also evaluated the use of centrifugal filter devices to recover DENV particles from non-infectious blood-meals presented to infected mosquitoes through a feeding membrane to collect their saliva.

## Introduction

Dengue fever is a mosquito-borne viral infection causing flu-like symptoms with potentially lethal complications most often in children. This disease circulates in many part of the world with continuous increasing geographic expansion and incidence [1]. Dengue fever is caused by one of the four serotypes of dengue virus (DENV), a member of the *Flavivirus* genus and *Flaviviridae* family. The transmission of DENV to a susceptible human host occurs through the bite of infected *Aedes* (*Ae*.) mosquito females, mainly *Ae*. *aegypti* [1,2]. DENV/vector interactions have been the subject of many studies [3–10]. The usual prerequisite to this kind of work is to infect mosquitoes with artificial blood-meals containing sufficient viral loads [9,10]. Moreover, retrieving the infectious saliva from DENV-infected mosquitoes may be necessary to establish viral transmission [5–8].

Concentration by ultrafiltration technique from culture supernatant has been successfully validated for retroviruses such as Moloney murine leukemia virus [11], pseudotyped HIV particles [12,13] and spleen necrosis virus [14] with a 30 to 250 fold gain in titer. For arboviruses, the use of ultrafiltration to concentrate DENV particles has been described in few publications in complement to ultracentrifugation for serological assays [15,16] and recently in a study on blood-products photochemical-treatment [17].

For the first time, we report the use of centrifugal filter devices (CFD) as a convenient and efficient method to concentrate DENV particles from infected cell-culture supernatants in the aim of preparing blood-meals for *per os* infection of mosquitoes. We also describe the interest of using CFD to concentrate DENV particles from non-infectious blood-meals presented to infected mosquitoes through a Parafilm-M membrane to collect their infectious saliva.

## Materials and Methods

### Cell line and Virus

*Ae*. *albopictus* clone C6/36 cells [18] (ATCC CRL-1660, USA) were routinely maintained at 30°C in cell-culture medium consisting in RPMI-1640 medium supplemented with non essential amino acids, gentamicin, fungizone (Amphotericin B) and 10% heat-inactivated foetal bovine serum (FBS, Life technologies, USA). The strain of DENV serotype-1 was isolated in French Polynesia in 2008 (PF08/080108-88).

### Amplification and concentration of virus particles

DENV was initially amplified from the serum by inoculation onto C6/36 cells at 1:40 in cell-culture medium adjusted to 1% of FBS. After 1 hour, the inoculum was removed and replaced with fresh 1% FBS-cell-culture medium. Infected cells were incubated at 30°C for 7 days and the cell-culture supernatant (SN-1P) was then collected and stored at –80°C after adding FBS at 1:5. For each CFD-concentration experiment, an aliquot of SN-1P was undergone three successive additional passages on C6/36 cells. Each passage was inoculated at 3:1 in 1% FBS-cell-culture medium for 2 hours at 37°C with a gentle agitation. The inoculum was then replaced by fresh 1% FBS-cell-culture medium and infected cells were incubated at 30°C for 4–5 days. At the conclusion of the fourth passage, the DENV-infected cell-culture supernatant (SN-4P) was centrifuged at 3,200 x g during 10 min at 4°C to be clarified from cells in suspension and pre-filtrated at 0.22 μm. The clarified SN-4P was loaded onto Centricon Plus-70 CFD (Millipore, Germany) and physically concentrated by centrifugation at 3,200 x g at 4°C. For a maximum recovery and given the 50 nm diameter of the mature DENV particle, we used devices with 10 nm membrane pore size (100K NMWL). The concentrate was recovered by inverting the filter device and centrifugation at 1,000 x g during 2 min. FBS was added at 1:5 before storage at -80°C.

### Virus titration

Samples of DENV-infected cell-culture supernatant were collected before and after concentration with CFD for titration of infectious viral particles by 96-wells plate assay in C6/36 cells. Titrations were performed by inoculation of 10-fold dilutions and 7 days after, by indirect immunofluorescent revelation. Briefly, C6/36 cells were fixed on the plates with cold acetone for 10 min and immunostained using DENV serotype 1 mouse hyperimmune ascitic fluid (ATCC VR71, USA) diluted 1:100 and then fluorescein isothiocyanate-conjugated sheep anti-mouse IgG (74641, Bio-Rad Laboratories, France) diluted 1:100. Infectious wells were counted and viral titers were expressed as 50% tissue culture infectious dose (TCID_50_/mL) using the method of Reed and Muench [19].

### Mosquito rearing

Eggs from laboratory-reared *Aedes aegypti* (Toahotu, Tahiti) were hatched under pressure for 1 hour. Larvae were reared in tap water supplemented with bovine liver powder (MP Biomedicals, USA) inside a climatic chamber (Sanyo MLR-351H, Japan) setting at 28°C, 80% humidity and a 12:12h light-dark cycle. Pupae were selected to adjust the gender ratio to 1:4 males. After emergence, mosquitoes were maintained in the climatic conditions indicated above and were given permanent access to 10% sugar solution.

### Mosquito infection

On the day of infection, five-days-old mosquitoes starved for 24 hours prior to the infection experiment were transferred into 70 x 54 mm cylindrical containers with nylon mesh on the top. The infectious meal was prepared with fresh washed bovine red cells, viral supernatant (1:2) and adenosine triphosphate at 5 mM as phagostimulant. Blood meal was offered through a Parafilm-M membrane and maintained at 37°C. After 20 min of free access to the blood meal, each fully-engorged female was transferred into an individual 67 x 26 mm plastic container to avoid horizontal transmission during sugar feeding. Indeed, expectoration of virus from infectious mosquitoes in the shared sugar-meal could infect mosquitoes that were not originally infected and leads to a bias in results [20,21]. Mosquitoes were held with 10% sugar solution for 14 days incubation period at 30°C prior to be sacrificed by freezing.

### Saliva collection

In three of the eight mosquito-infection trials performed, mosquitoes were pooled by twenty into a 70 x 54 mm container at the day before the end of the 14 days-incubation period and starved for 24 hours. Non-infectious blood-meals were then simultaneously offered to the pools of mosquitoes for 1 hour, allowing them to probe, take blood and excrete saliva within the meal. The blood-meals containing the infectious saliva were centrifuged at 20,000 x g during 3 min at 4°C to pellet the red cells. The meal supernatants were then all pooled together in one single sample. Concentration was performed using Amicon Ultra-2 CFD (100K NMWL; Millipore, Germany) by centrifugation at 2,000 x g at 4°C until the retentate achieved an appropriate volume for subsequent RNA extraction. The mosquito-saliva DENV retentate was recovered by inverting the filter device and centrifugation at 1,000 x g for 2 min. FBS was added at 1:5 before storage of the retentate at -80°C.

### RNA extraction and reverse transcription polymerase chain reaction (RT-PCR) detection

Mosquitoes were individually homogenized with metal beads at 20 Hz for 4 min (Mixer Mill Retsch MM301, Germany) in cell-culture medium supplemented at 20% FBS. Each mosquito homogenate was then centrifuged at 20,000 x g for 5 min at 4°C and supernatant was recovered. DENV RNA was extracted from the mosquito supernatant and the mosquito-saliva retentate using QIAamp RNA mini kit (Qiagen, Australia) following manufacturers’ instructions. DENV real-time TaqMan RT-PCR were performed on the iCycler iQ Detection System instrument with the iScript One-Step RT-PCR Kit for Probes (Bio-Rad Laboratories, France) as previously described [22,23]. A standard curve using serial dilutions of total RNA was included to estimate the copy number of DENV RNA obtained in mosquito-saliva retentate.

### Data and statistical analysis

Viral titers were log_10_ transformed and checked for normality using Shapiro-Wilk test. Paired t-test was used to assess the difference between viral titers of the cell-culture supernatants before and after concentration with CFD (Graph Pad Prism software, USA).

## Results

### Use of CFD to obtain DENV concentrates from C6/36 cell-culture supernatants

**Fig 1:**
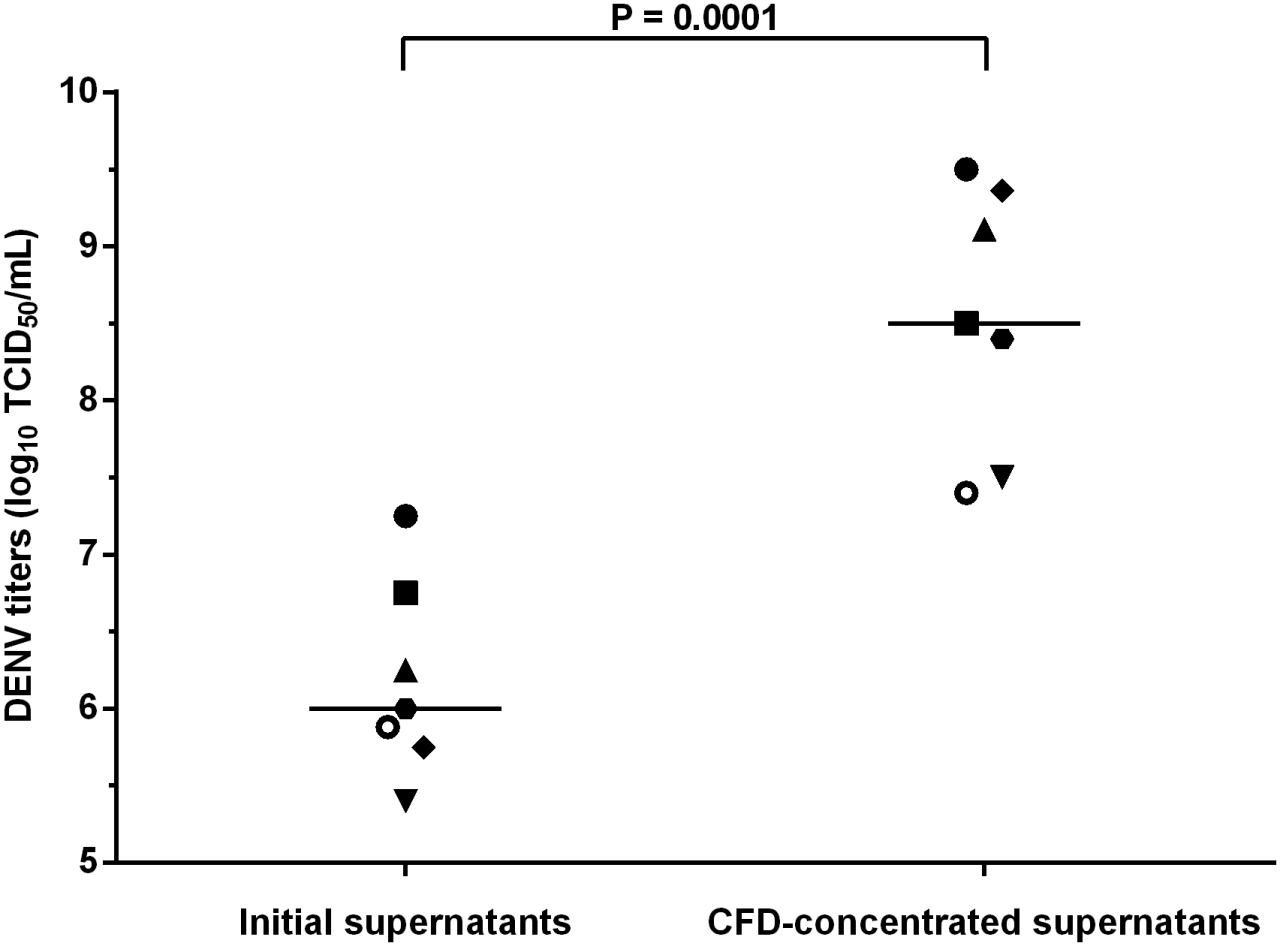
Effect of using centrifugal filter device on DENV titers in C6/36 cell-culture supernatants. DENV titers in C6/36 cell-culture supernatants were evaluated before and after concentration with Centricon Plus-70 centrifugal filter devices (CFD; Millipore, Germany). Viral titers were significantly higher after CFD concentration (Paired t-test, P = 0.0001) with an evolution of the median titer (line) from 6.00 to 8.50 log_10_ TCID_50_/mL. DENV = Dengue virus.

Seven experimental assays were performed to concentrate DENV particles from cell-culture supernatants. For each experimental assay, a volume of 280 mL of SN-4P was concentrated to obtain approximately 1mL of concentrate in less than an hour. The DENV titers in infected cell-culture supernatants before CFD concentration ranged from 5.40 to 7.25 log_10_ TCID_50_/mL with a median titer at 6.00 log_10_ TCID_50_/mL (Fig 1). After CFD concentration, the viral titers were significantly higher (p = 0.0001) with values between 7.40 and 9.50 log_10_ TCID_50_/mL and a median titer at 8.50 log_10_ TCID_50_/mL (Fig 1). The mean gain was 2.36 ± 0.27 log_10_ (mean ± standard error), gain representing the viral titer in the CFD-concentrated supernatant subtract the viral titer in the supernatant before CFD-concentration.

### Infection rate obtained in *Aedes aegypti* with the use of CFD-concentrated DENV

Eight independent experimental *per os* infections of mosquitoes were performed using CFD-concentrated DENV. The infection rates calculated as the number of DENV RT-PCR-positive mosquitoes divided by the number of mosquitoes tested are shown in Table 1. The purpose of trial 1 was to check that performing CFD concentration on a supernatant with a very low initial DENV titer will allow increasing the viral titer in the blood meal and result in higher mosquito infection rates. Using CFD-concentrated supernatant, the titer in the blood meal increased from 5.4 (trial 1a) to 6.92 (trial 1b) log_10_ TCID_50_/mL and mosquito infection rate increased from 38.89% (trial 1a) to 92.86% (trial 1b). No excessive mortality was observed when the mosquitoes were fed with CFD-concentrated DENV (4.5%, trial 1b) versus initial supernatant (5.26%, trial 1a). Trials 2 to 8 were designed to demonstrate that CFD-concentration of supernatants with various initial DENV titers allowed repeatedly preparing blood meals with viral titers over 7 log_10_ TCID_50_/mL what repeatedly resulted in high mosquito-infection rates (>86%, mean rate of 94 ± 1.77%.).

**Table 1:** Infected *Aedes aegypti* rate (%) at 14 days post-intake of an artificial infectious blood-meal prepared either with initial (trial 1a) or CFD-concentrated DENV supernatants (trials 1b-8).

Experimental assay	DENV titer in blood meals (log_10_ TCID_50_ /mL)	% of infected mosquitoes (N)	DENV RNA load in saliva retentates (log_10_ copies /μL)
Trial 1a	5.40	38.89 (18)	-
Trial 1b	6.92	92.86 (42)	-
Trial 2	7.02	86.67 (30)	-
Trial 3	8.64	90 (20)	-
Trial 4	8.89	100 (27)	-
Trial 5	8.89	100 (37)	-
Trial 6	7.92	95.56 (45)	4.26
Trial 7	7.92	91.07 (56)	4.42
Trial 8	7.92	98.65 (74)	5.13

The table indicates the DENV titers in the blood-meals at day 0, the percentages (%) of DENV-positive mosquitoes obtained 14 days later and for the three last trials, the copy numbers of DENV RNA obtained in the saliva retentates from the infected-mosquitoes expectoration within non-infectious blood-meals at day 14. DENV = Dengue virus. N = number of RT-PCR-tested mosquitoes. A dash (-) indicates that saliva was not collected for these trials.

### Use of CFD to retrieve DENV from *Aedes aegypti* saliva excreted within artificial blood meals

In the last three trials, the infected mosquitoes were allowed at the end of the 14 days-incubation period to excrete saliva within usual non-infectious blood-meals before their sacrifice. Obtained blood-meal supernatants were all pooled together in one single sample and concentrated through the use of CFD to an appropriate volume for RNA extraction. DENV was detected in the three saliva retentates with a copy number of RNA of at least 4.26 log_10_ copies per μL (Table 1).

## Discussion

Virus/vector studies commonly require the infection of mosquitoes [3–10]. Viral loads obtained in cell-culture supernatants can be insufficient to efficiently infect mosquitoes through artificial blood-meals. Several methods to concentrate viral particles exist, notably polyethylene glycol precipitation, pressurized filtration and ultracentrifugation over a density gradient [11–16]. However, these methods are time-consuming and a significant loss in infectivity can appear [11,12]. In the present study we described the use of CFD to concentrate infectious DENV particles in cell-culture supernatants by ultrafiltration with no more need for further ultracentrifugation as usually performed [15,16]. These CFD-concentrated supernatants were then evaluated in mosquito *per os* infection experiments. CFD-concentration of DENV cell-culture supernatants provided in less than one hour and no need for particular reagents or sophisticated equipment except a regular centrifuge, a mean gain of 2.36 log_10_ TCID_50_/mL in DENV titer. CFD-concentrated supernatants were then used to prepare highly infectious blood-meals that resulted in a mean rate of mosquito infection of 94%.

Retrieving virus from saliva of infectious mosquitoes can be necessary to assess transmission of virus [5–8]. Several methods have been described to collect saliva from mosquitoes such as blood-droplets [10], sugar-impregnated gauze pledgets [21] and mostly forced salivation [5–8,24–26]. In the present study, we show for the first time the possibility to use the blood-feeding membrane system to collect DENV from mosquito-saliva. During blood intake, infectious mosquitoes expectorated saliva containing DENV within the blood-meal. The use of CFD allowed concentrating large volumes of blood-meal to an appropriate volume for RNA extraction. The recovery of DENV from saliva expectorated by infected mosquitoes within blood-meals was validated by positive RT-PCR and the transcript copy number values obtained were consistent to those previously published using forced-salivation commonly used [5–8,24]. Furthermore, this method allows the advantageous possibility to collect saliva from the same pool of mosquitoes on successive days in contrast to forced-salivation in which legs and wings are removed and mosquito sacrifice is finally required [25,26].

## Conclusions

We evaluated for the first time the efficiency of using ultrafiltration technique to concentrate DENV in a context of virus/vector studies. Using blood-meals prepared with CFD-concentrated DENV in *per os* infection experiments of *Ae*. *aegypti*, we obtained high mosquito-infection rates. We also describe the use of CFD as an efficient method to concentrate DENV from blood-meals presented through a feeding membrane to collect the infectious saliva of infected-mosquitoes.
